# The power of patient stories: stewardship and *C. difficile* prevention

**DOI:** 10.1017/ash.2026.10309

**Published:** 2026-03-03

**Authors:** Denise Cardo, Christian John Lillis

**Affiliations:** 1 Retired, Centers for Disease Control and Prevention, Atlanta, USA; 2 Peggy Lillis Foundation, USA

In healthcare, data guide our decisions—but patient stories drive our mission and remind us why the work matters.

The United States has made significant strides in *Clostridioides difficile* infection (CDI) prevention through improved diagnostics, antimicrobial stewardship, and infection control. Yet progress remains uneven, and preventable harm persists. When we speak only in data, patient’s suffering risks becoming background noise. Patient stories interrupt that process. They remind us that no case is routine, no infection is merely a metric, and no family is untouched by loss when prevention fails.

The Peggy Lillis Foundation exemplifies how storytelling can catalyze change. What began as one family’s grief evolved into organized advocacy that has influenced public awareness, clinician education, and policy dialogue. In parallel, CDC efforts under Dr. Denise Cardo increasingly incorporated lived experience into patient safety initiatives, recognizing that survivors and caregivers bring expertise that surveillance systems alone cannot provide.

To fully leverage the power of narrative, we must commit to ethical storytelling. Stories should not compete with data but augment it—placing people at the center of stewardship and prevention work.

## How have individual patient narratives—like Peggy’s—led to tangible shifts in policy, CDC priorities, or institutional practices that data alone could not achieve?

Denise Cardo (DC): I believe that where we are today in terms of prevention impact is because of patient engagement. For example, the push for public reporting of infections and accountability grew out of families demanding transparency and legislative action after devastating outcomes.

At the time, Lisa McGiffert at *Consumer Reports* played a critical role in advancing state-level reporting. Many of us were initially skeptical, thinking, “No one will understand the data.” But it quickly became clear that this was the path forward. Either we would be part of the movement, or change would occur without the expertise of healthcare epidemiology organizations, like CDC and SHEA.

That realization prompted a major shift. CDC’s Healthcare Infection Control Practices Advisory Committee (HICPAC) developed guiding principles for public reporting, and SHEA collaborated with Lisa and others to draft state legislation. This was transformative. Previously, we were often perceived as resistant to accountability. We learned that partnering with families who had lost loved ones, or patients who survived severe infections, was essential to transparency, accountability, and ultimately, the goal of elimination.

For CDC, this marked a move from benchmarking to active prevention. We had to learn how to present data in ways that were understandable and actionable at every level. The 2010 CDC–SHEA–APIC–IDSA International Decennial Conference adopted the theme “Moving Toward Elimination,” a shift that would not have been possible without patient advocates.

Christian John Lillis (CL): Lisa connected me with Denise and the team at CDC’s Division of Healthcare Quality Promotion. Historically, we’ve seen patient stories catalyze systemic change—whether through the Ryan White HIV/AIDS Program or Susan G. Komen’s work in breast cancer advocacy.

I believe patient stories, including my mother’s, were instrumental in advancing antibiotic stewardship programs nationwide during the 2010s. I spoke frequently about this and contributed to the Antibiotic Stewardship Playbook. Between 2012 and 2019, CDC regularly convened patient advocates to meet with DHQP staff and leadership.

These meetings led to deep—and sometimes uncomfortable—conversations. Over time, we saw CDC increasingly center patients in both internal culture and external communications. This also helped patient advocates better understand the scope and limits of these agencies. People often say, “CDC should outlaw X,” but CDC is an advisory body, not a regulatory body. Pop culture depictions—like *Outbreak*—don’t reflect that reality.

These interactions also shaped our approach to advocacy. During “Lobby days” with groups like IDSA, SHEA, and Pew, legislators needed to hear from patients alongside clinicians and scientists about why sustained funding for CDC and NIH matters—especially in moments like the one we’re in now.

An underappreciated area is the role of patient voices in drug approvals and label expansions, particularly for treatments that aren’t blockbuster products. Patients and families learn from this work and then advocate for access to appropriate therapies for infections.

## We often cite incidence, mortality, and recurrence rates for *C. difficile*. What gets lost when we speak in data points rather than people?

DC: When I was at CDC, I often opened or closed presentations with photos of people who had survived or died from infections. Those images conveyed the urgency of prevention in a way numbers cannot. Stories and faces are the motivation for our work.

That’s why we regularly invited patient groups to CDC to meet with experts, including the CDC Director. It reinforced the importance of engaging patients as partners in safety efforts.

CL: There’s an old saying: “One death is a tragedy; a million is a statistic.” Humans can’t emotionally process loss at scale. We saw this during COVID-19—people became numb or disengaged. That emotional disconnect creates fertile ground for misinformation.

We’re storytelling creatures. When we hear that someone’s grandparent died, we think of our own. That’s tangible in a way that “500,000 cases” never is. Part of the Peggy Lillis Foundation’s impact is that my mother’s story—her life as a kindergarten teacher and a single mom—was relatable. Her death from antibiotic misuse and lack of awareness of *C. difficile* reflects a scenario many people recognize in our healthcare system.

Stories become an entry point into the data. They help people grasp the magnitude of societal loss behind the statistics.

DC: I recently heard someone say that “stories and faces are data with tears.”

## Where is the line between honoring lived experience and unintentionally exploiting trauma?

CL: We emphasize respecting boundaries. Telling my mother’s story still forces me to relive the worst 36 hours of my life, but I do it because it’s necessary—and because I know my limits. We train our speakers to do the same.

Organizations should do their homework. If a story is publicly available, review it before asking someone to retell it. Avoid invasive or unrelated questions, especially about litigation or graphic details. Don’t ask about how children or family members are coping unless the speaker raises it themselves.

Another critical principle is compensation. Never ask patient advocates to pay out-of-pocket for travel or lodging. Many are dealing with financial strain related to illness or loss. Paying them recognizes their expertise and value. And remember that patient advocates don’t need this on their CVs. They’re doing it to prevent harm and influence change.

DC: From an organizational perspective, the first principle is to listen to learn—not to respond or justify. Families are often angry, and rightly so. They don’t want explanations; they want to be heard.

I remember one of the first families we met at CDC, who were extremely angry. We listened. We didn’t argue or minimize their experience. That changed everything. The conversation shifted toward what we could do together so no other families would endure the same loss. They became some of our fiercest partners.

Most families affected by preventable harm aren’t seeking compensation. They want meaning to come from their pain. Many have created foundations or advocacy efforts that have transformed prevention policy.

## How do we ensure patient testimonies elevate—rather than compete with—scientific truths?

DC: They don’t compete if trust is established and information is shared consistently. Patient advocates should be at the table from day one—not after decisions are made.

CL: Institutions like NIH and FDA have historically kept the public at arm’s length, which makes them vulnerable to misinformation campaigns and bad-faith attacks. Transparent, respectful dialogue builds trust.

During COVID-19, clearer communication about uncertainty and evolving evidence could have prevented some backlash. Instead, institutions created ambiguity that others exploited.

A current example involves fecal microbiota transplant (FMT). When FDA ended enforcement discretion after approving new microbiota products, OpenBiome could no longer operate—even though many centers still relied on it. Now, access to FMT is extremely limited, which is specifically harmful for pediatric patients who aren’t yet eligible for newer microbiota products. Even if families can find an FMT provider, they are often forced to travel and pay thousands of dollars out of pocket.

When my mother died in 2010, FMT wasn’t widely available. Today, we have FDA-approved microbiome therapies—but access remains inequitable. Regulatory agencies must prioritize patient and public interest above all else.

## What progress are you most proud of in patient- and family-driven policy change?

CL: First, public awareness. In 2010, only about 25% of Americans had heard of *C. difficile*. Today, that number is closer to 46%. Campaigns like “See C. Diff” have reached audiences traditional research channels often miss.

Second, while it hasn’t yet passed, the introduction of the Peggy Lillis *C. difficile* Inclusion Act—which would make *C. difficile* nationally notifiable—was a profound moment. When I’m exhausted or discouraged, I ask myself, “What would my mother do?” That keeps me going.

DC: Public reporting at state and federal levels is a major achievement, as is including patient representation on HICPAC, other CDC advisory committees, and national conferences.

We’ve seen similar impact in sepsis advocacy, driven by families like Rory Staunton’s and organizations such as the Sepsis Alliance. These efforts show how loss can be transformed into accountability and prevention across multiple conditions.

CL: I hope we can sustain this momentum despite the current political challenges. Denise’s mentorship—and the culture she fostered at CDC—has been extraordinary. Although some colleagues have moved on from the CDC, I remain hopeful.

DC: We must continually remind ourselves why we do this work. That’s how we move from darkness toward purpose—and toward saving lives.

